# Automatic Segmentation of the Left Ventricle in Apical Four-Chamber View on Transesophageal Echocardiography Based on UNeXt Deep Neural Network

**DOI:** 10.3390/diagnostics14232766

**Published:** 2024-12-09

**Authors:** Lingeer Wu, Yijun Ling, Ling Lan, Kai He, Chunhua Yu, Zhuhuang Zhou, Le Shen

**Affiliations:** 1 Department of Anesthesiology, Peking Union Medical College Hospital, Chinese Academy of Medical Sciences and Peking Union Medical College, Beijing 100730, China; 13426365656@126.com (L.W.); lanling_1988@163.com (L.L.); harveyhekai@sina.com (K.H.); yuchh@pumch.cn (C.Y.); 2 Department of Biomedical Engineering, College of Chemistry and Life Science, Beijing University of Technology, Beijing 100124, China; lingyijun@emails.bjut.edu.cn (Y.L.); zhouzh@bjut.edu.cn (Z.Z.)

**Keywords:** transesophageal echocardiography, left ventricle, segmentation, deep learning

## Abstract

**Background/Objectives:** The automatic left ventricle segmentation in transesophageal echocardiography (TEE) is of significant importance. In this paper, we constructed a large-scale TEE apical four-chamber view (A4CV) image dataset and proposed an automatic left ventricular segmentation method for the TEE A4CV based on the UNeXt deep neural network. **Methods:** UNeXt, a variant of U-Net integrating a multilayer perceptron, was employed for left ventricle segmentation in the TEE A4CV because it could yield promising segmentation performance while reducing both the number of network parameters and computational complexity. We also compared the proposed method with U-Net, TransUNet, and Attention U-Net models. Standard TEE A4CV videos were collected from 60 patients undergoing cardiac surgery, from the onset of anesthesia to the conclusion of the procedure. After preprocessing, a dataset comprising 3000 TEE images and their corresponding labels was generated. The dataset was randomly divided into training, validation, and test sets in an 8:1:1 ratio on the patient level. The training and validation sets were used to train the UNeXt, U-Net, TransUNet, and Attention U-Net models for left ventricular segmentation. The dice similarity coefficient (DSC) and Intersection over Union (IoU) were used to evaluate the segmentation performance of each model, and the Kruskal–Wallis test was employed to analyze the significance of DSC differences. **Results:** On the test set, the UNeXt model achieved an average DSC of 88.60%, outperforming U-Net (87.76%), TransUNet (85.75%, *p* < 0.05), and Attention U-Net (79.98%; *p* < 0.05). Additionally, the UNeXt model had a smaller number of parameters (1.47 million) and floating point operations (2.28 giga) as well as a shorter average inference time per image (141.73 ms), compared to U-Net (185.12 ms), TransUNet (209.08 ms), and Attention U-Net (201.13 ms). The average IoU of UNeXt (77.60%) was also higher than that of U-Net (76.61%), TransUNet (77.35%), and Attention U-Net (68.86%). **Conclusions:** This study pioneered the construction of a large-scale TEE A4CV dataset and the application of UNeXt to left ventricle segmentation in the TEE A4CV. The proposed method may be used for automatic segmentation of the left ventricle in the TEE A4CV.

## 1. Introduction

Echocardiography is a non-invasive medical imaging technique and serves as a primary method for the clinical dynamic assessment of cardiac function. It can be divided into two categories: transthoracic echocardiography (TTE) and transesophageal echocardiography (TEE). By analyzing echocardiographic images, multiple important cardiac function parameters can be quantitatively obtained, with accurate segmentation of the left ventricle serving as the foundation for subsequent metric calculations. Currently, clinicians typically rely on manual segmentation of the left ventricular contours from echocardiography, a process that is time-consuming and labor-intensive, and heavily dependent on the clinician’s personal experience. The measurements of the same patient may exhibit inter-operator differences as well as intra-operator differences. Deep learning techniques have demonstrated significant potential in medical image analysis, offering effective solutions for automatic segmentation of the left ventricle in echocardiography.

In the past decade, advancements in deep learning-based semantic segmentation methods, such as fully connected networks (FCNs) [1], DeepLab [2,3,4], and U-Net [5,6,7,8], have facilitated their increasing applications in medical image segmentation. DeepLab has three versions: V1 [2], V2 [3], and V3 [4]. DeepLab V1 incorporates atrous convolution and conditional random fields (CRFs), using VGG16 as the backbone [2]. DeepLab V2 further incorporates atrous spatial pyramid pooling (ASPP) with ResNet-101 as the backbone [3]. DeepLab V3 builds on DeepLab V2 and uses improved ASPP and deeper atrous convolution while removing CRFs [4]. The U-Net series, including U-Net [5], UNet++ [6], nnU-Net [7], and UNet3+ [8], have shown promising performance in medical image segmentation. Recently, researchers have combined attention mechanisms with convolutional neural networks (CNNs) for medical image segmentation to enhance the model’s global perception capabilities. Notable models include DANet [9], CBAM [10], and Attention U-Net [11]. Additionally, the advantages of Transformers in capturing long-range dependencies have led to their integration with CNNs, resulting in models like TransUNet [12], Swin Transformer [13], and CvT [14]. Researchers have improved network architectures based on the aforementioned methods for TTE left ventricular segmentation tasks. Liu et al. [15] designed a U-Net-based model, DPS-Net, achieving a dice similarity coefficient (DSC) of 93.2% (end diastole) and 92.8% (end systole) on the CAMUS (Cardiac Acquisitions for Multi-structure Ultrasound Segmentation) dataset [16]. Moradi et al. [17] proposed MFP-U-Net, which enhanced U-Net’s skip connection structures by merging features from each layer of the decoder with those of the encoder, achieving a DSC of (94.5 ± 0.12)% on the CAMUS dataset. Zeng et al. [18] proposed the MAEF-Net, which employed multiple attention mechanisms to capture heartbeat features while suppressing noise, enabling fully automated left ventricle segmentation. Their model achieved a DSC of (93.1 ± 2.22)% on the EchoNet-Dynamic dataset [19]. Zhu et al. [20] presented SAM-Att, which integrated an attention module into the segment anything model (SAM), resulting in a DSC of 93.49% on the CAMUS dataset. Lal et al. [21] enhanced U-Net by incorporating residual modules and modifying the skip connection structure, achieving a DSC of 95.59% on the CAMUS dataset for left ventricular segmentation. Wan et al. [22] employed a semi-supervised learning approach for left ventricular segmentation, attaining DSCs of 93.05% and 93.93% on the CAMUS and EchoNet-Dynamic datasets, respectively.

Although deep learning techniques have been extensively studied for automatic left ventricle segmentation in the apical four-chamber view (A4CV) on TTE, demonstrating the desired performance [15,17,18,20,21,22], research on deep learning methods for segmenting the left ventricle in the TEE A4CV remains limited. Few studies involve applications of deep learning in automatic left ventricular segmentation in TEE. Kang et al. [23] introduced an attention mechanism and a residual feature aggregation module in U-Net for segmenting the left ventricle in the long-axis view of the mid-esophagus, achieving a DSC of (89.9 ± 1.7)%. However, the dataset was too small, containing only 120 TEE images from nine patients. Ahn et al. [24] employed Res-U-Net++ [25], combining features extracted from 158 TEE images (number of patients not described) for left ventricular segmentation, resulting in a DSC of 82.08%. The small number of images in existing deep learning methods on left ventricle segmentation in TEE may limit their further development. Moreover, the current deep learning models for left ventricular segmentation in TEE had large parameter sizes, which may lead to longer inference times. Therefore, it may be needed to construct a large-scale TEE image dataset. In addition, UNeXt [26] is a recently introduced deep neural network based on U-Net, which shows promising performance in image segmentation with a reduced number of network parameters. However, the UNeXt [26] model has not been applied to TEE image segmentation.

In this paper, we constructed a large-scale TEE A4CV image dataset, as there is no publicly available dataset for TEE A4CV, and proposed an automatic segmentation method for the left ventricle in the TEE A4CV based on the UNeXt [26] deep neural network. UNeXt uses U-Net as the backbone network and incorporates a multilayer perceptron (MLP), which could produce promising segmentation performance while reducing both the number of network parameters and computational complexity. Furthermore, UNeXt has not been applied to left ventricular segmentation in the TEE A4CV. Standard TEE A4CV videos were collected from 60 patients undergoing cardiac surgery, from the onset of anesthesia to the conclusion of the procedure. After data preprocessing, a dataset containing 3000 TEE images and corresponding labels was generated. The dataset was randomly divided into training, validation, and test sets, ensuring that images from the same patient appeared in only one subset. U-Net [5], TransUNet [12], and Attention U-Net [11] were utilized for comparison. The training and validation sets were input into the UNeXt, U-Net, TransUNet, and Attention U-Net networks to train left ventricular segmentation models. The test set was used to evaluate the segmentation performance of each model. The experimental results indicated that the proposed method improved segmentation accuracy while simultaneously reducing the number of network parameters and inference time.

## 2. Materials and Methods

**Figure 1:**
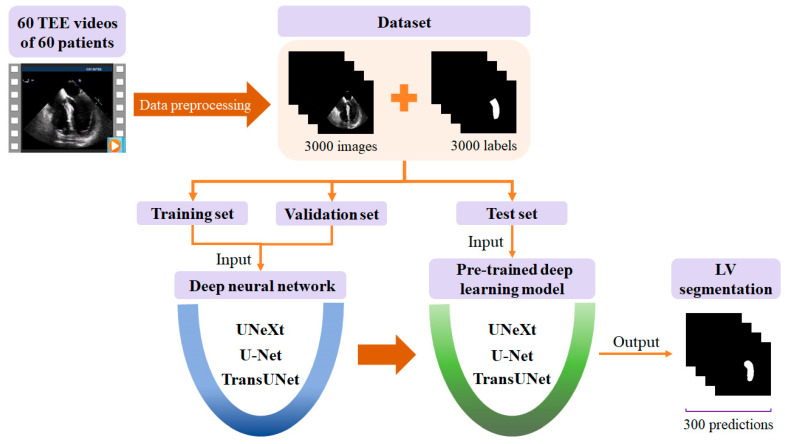
Flow chart of the proposed deep learning-based method for automatic segmentation of the left ventricle (LV) in the transesophageal echocardiography (TEE) apical 4-chamber view.

Figure 1 illustrates the process of automatic segmentation of the left ventricle in the TEE A4CV. Initially, a dataset containing 3000 TEE images and their corresponding labels was obtained from the TEE videos of 60 patients after data preprocessing. The dataset was randomly divided into training, validation, and test sets in an 8:1:1 ratio. The training and validation sets were used to train and optimize the model. Subsequently, the test set was input into the trained deep learning network model to obtain 300 segmented images of the left ventricle.

### 2.1. TEE Dataset

#### 2.1.1. Data Collection

**Figure 2:**
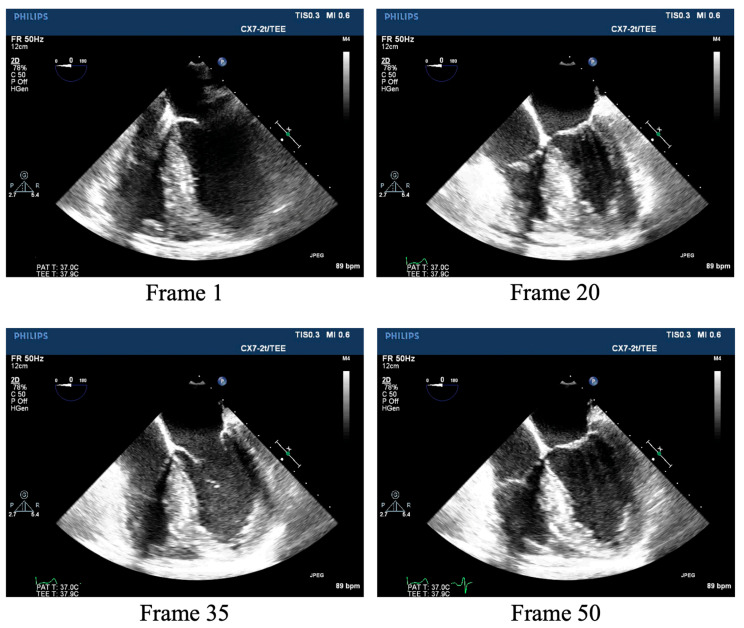
The 1st, 20th, 35th, and 50th TEE A4CV frames of a TEE video of the same patient. TEE: transesophageal echocardiography; A4CV: apical four-chamber view.

The TEE data were collected from the Department of Anesthesiology at Peking Union Medical College Hospital, comprising standard TEE A4CV videos from 60 patients undergoing cardiac surgery. The videos were acquired from the onset of anesthesia to the completion of the procedure. This retrospective, single-center study was approved by the Ethics Review Committee of the Peking Union Medical College Hospital, Chinese Academy of Medical Sciences. The video resolution was 800 × 600 pixels, with each video containing at least 1–1.5 cardiac cycles (Figure 2). The ultrasound videos were acquired by two professional anesthesiologists using an ultrasound scanner (IE33; Philips Medical Systems, Andover, MA, USA), with an X7-2t 1.0−5.0 MHz transducer (Philips Medical Systems). Note that TEE acquisition was much more difficult than TTE scanning because it involved the intervention of the ultrasound transducer.

#### 2.1.2. Data Preprocessing

**Figure 3:**
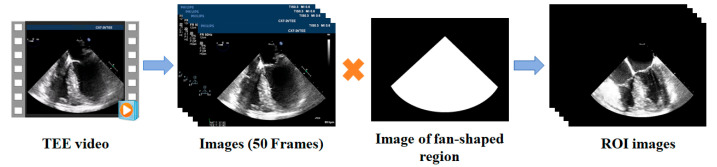
Generation of ROI images. TEE: transesophageal echocardiography; ROI: region of interest.

**Figure 4:**
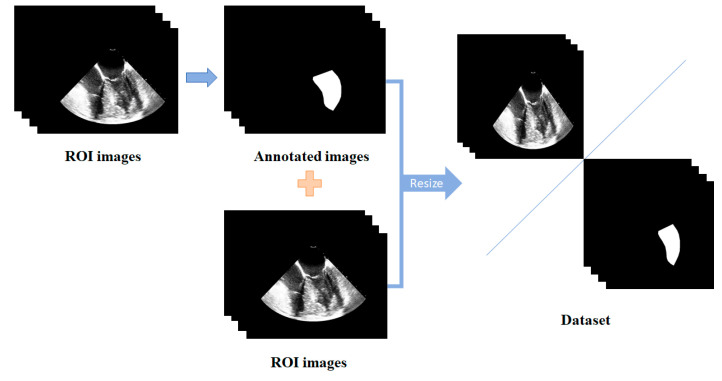
Process of constructing the dataset. ROI: region of interest.

For each TEE video, 50 consecutive frames covering at least one complete cardiac cycle were selected. As shown in Figure 3, a binary mask with a fan-shaped region (center: (425.36, 106), radius: 494 pixels, angle: 92.67°) was multiplied with each frame of the images to remove patient identifiers, scanning details, and other irrelevant information, which might interfere with left ventricle segmentation. This resulted in 50 ultrasound images of the region of interest (ROI) for each video (Figure 3). All ROI ultrasound images were manually annotated using the open access LabelMe software (version 4.5.13) to generate corresponding binary masks for the left ventricle (Figure 4). Each binary mask was stored as a “.json” file corresponding to the TEE A4CV image. The manual annotation was performed by an anesthesiologist and checked by another anesthesiologist, both of whom were experienced in the TEE A4CV. To meet the input requirements of the convolutional neural network (CNN), all grayscale ROI ultrasound images and their corresponding label masks were resized to 512 × 512 pixels using bilinear interpolation. These resized images were then used to form the TEE dataset (Figure 4). The TEE dataset was randomly divided into three subsets in an 8:1:1 ratio, a training set of 48 patients (2400 images), a validation set of 6 patients (300 images), and a test set of 6 patients (300 images), ensuring that images from the same patient appeared only in one subset. The training set was utilized to train the deep learning model, the validation set was used to monitor the model and adjust its optimal parameters, and the test set was used to evaluate the model’s segmentation performance.

### 2.2. Deep Learning Network Models

**Figure 5:**
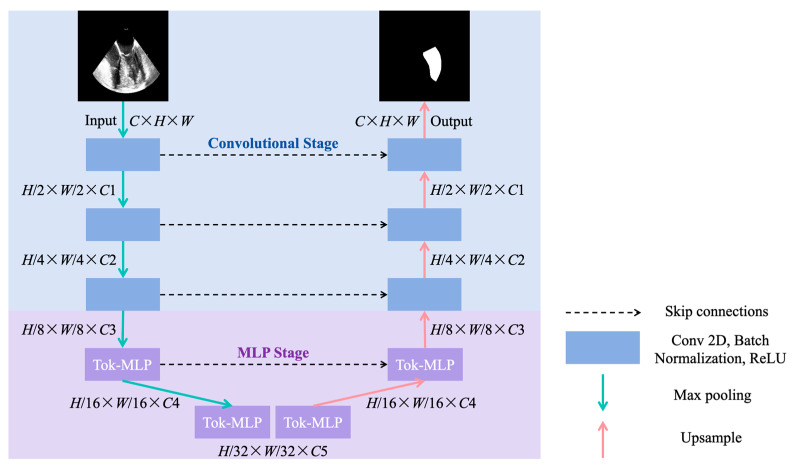
Structure of UNeXt [26]. (*C*, *H*, *W*) represent the channels, height, and width of the TEE image, respectively. Conv: convolutional; ReLU: rectified linear unit; MLP: multilayer perceptron; Tok-MLP: tokenized multilayer perceptron; TEE: transesophageal echocardiography.

In this study, UNeXt [26] was selected as the model for left ventricular segmentation in TEE (Figure 5). UNeXt uses U-Net as the backbone network and incorporates an MLP. The overall structure of UNeXt follows a U-shaped encoder–decoder architecture. The first three layers of the encoder consist of convolutional layers, which perform three downsampling operations on the input image. The fourth and fifth layers are MLP modules, where a Shifted-MLP module is introduced before a tokenized MLP (Tok-MLP) module. The Shifted-MLP alters channels before passing them into the MLP to focus the network on learning local features. The Tok-MLP module reduces the number of network parameters and computational complexity by using tokenized MLPs, while incorporating shift operations to extract local information across different axes for better feature representation. A skip connection structure is employed between the encoder and decoder, allowing the model to utilize features at different scales simultaneously. UNeXt can yield satisfying segmentation performance while reducing the number of network parameters, thereby lowering computational costs.

**Figure 6:**
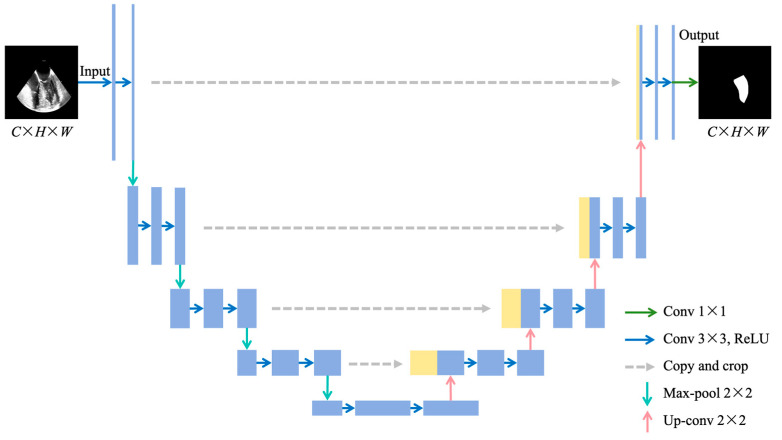
Structure of U-Net. (*C*, *H*, *W*) represent the channels, height, and width of the TEE image, respectively. Conv: convolutional; ReLU: rectified linear unit; TEE: transesophageal echocardiography.

As comparison methods, this study also selected U-Net [5], TransUNet [12], and Attention U-Net [11] for TEE left ventricular segmentation. U-Net is a CNN architecture widely used for medical image segmentation tasks. It features a U-shaped network structure composed of an encoder, a decoder, and skip connections (Figure 6). In the encoder, the input image undergoes four convolutional blocks and four max-pooling operations, extracting feature information and reducing spatial resolution. After the fifth convolutional block, the decoder performs four upsampling operations to gradually reconstruct the feature maps extracted by the encoder, restoring them to the original image resolution. The skip connections transfer feature maps from the encoder to the decoder to retain spatial information that may have been lost during downsampling.

**Figure 7:**
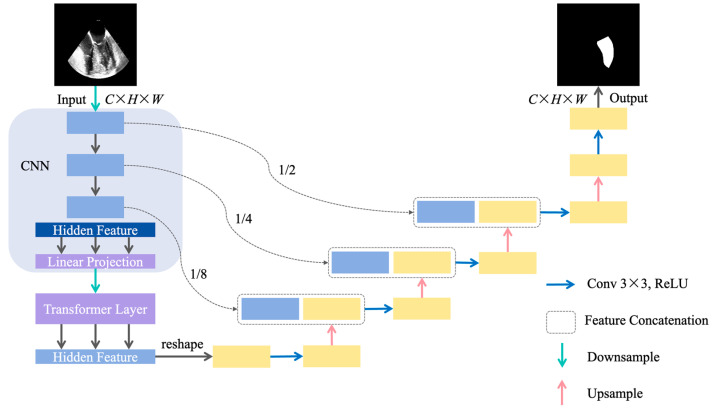
Structure of TransUNet [12]. (*C*, *H*, *W*) represent the channels, height, and width of the TEE image, respectively. Conv: convolutional; ReLU: rectified linear unit; TEE: transesophageal echocardiography.

TransUNet [12] builds on the U-Net framework and introduces an attention mechanism, specifically the self-attention module, to better capture global information (Figure 7). The overall structure of TransUNet is also U-shaped, with an encoder–decoder architecture. The encoder first splits the input TEE image into fixed-size patches, flattens these patches into one-dimensional vectors, and uses a linear transformation to map each vector into a high-dimensional space, forming feature vectors. Positional encoding is then applied, adding the positional encoding vector to each feature vector to retain spatial location information. The self-attention mechanism in the Transformer layers captures global features and long-range dependencies of the feature vectors. The upsampling operations in the decoder restore the sequence data output by the encoder to the original image size, while skip connections leverage features at different scales.

**Figure 8:**
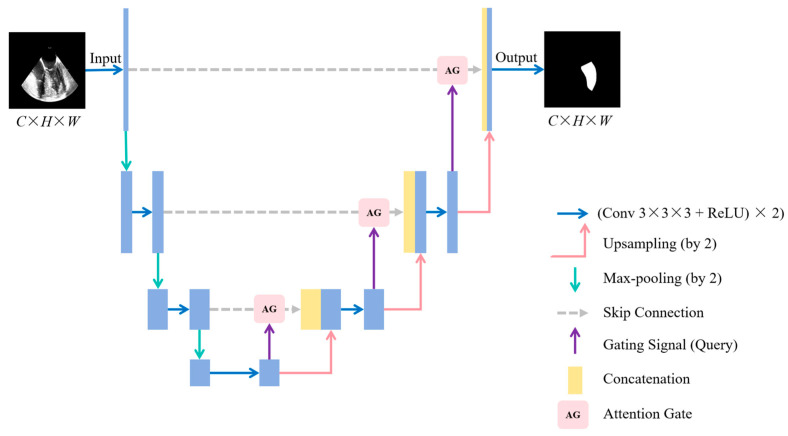
Structure of Attention U-Net. (*C*, *H*, *W*) indicates the channels, height, and width of the TEE image. Conv: convolutional; ReLU: rectified linear unit; TEE: transesophageal echocardiography.

Attention U-Net [11] is based on the U-Net architecture, with the addition of an attention gate to enhance the representation of features in regions of interest. The network consists of an encoder, a decoder, and skip connections (Figure 8). The encoder, similar to that of U-Net, comprises convolutional layers and max-pooling layers. An attention gate is employed within the skip connections, enabling the fusion of low-level encoder features with high-level decoder features. The generated attention weights are applied to reweight the features, suppressing irrelevant background information and emphasizing target features.

### 2.3. Experimental Setup

The experiment was conducted on a workstation configured with two Intel (R) Xeon (R) Gold 6132 CPUs @ 2.60 GHz, an NVIDIA TITAN RTX 24 G GPU, and 128 GB of RAM. The deep learning framework used Python (version 3.6) and PyTorch (version 1.10). In the experiments, the batch size was set to 16, and the training process was run for 100 epochs. The Adam optimizer (adaptive moment estimation) was employed, with an initial learning rate (LR) of 10^−3^ and a minimum LR of 10^−5^. The learning rate adjustment followed a cosine annealing strategy (CosineAnnealing LR), and the momentum was set to 0.9. The input dimensions for the model were set to (*C* × *H* × *W*) = (3 × 512 × 512), where *C*, *H*, and *W* represent the number of channels, height, and width of each frame, respectively. The output dimensions were (*C* × *H* × *W*) = (1 × 512 × 512).

The binary cross-entropy loss (*L*_BCE_) function was selected as the loss function for the experiments. Binary cross-entropy was used to measure the difference between the predicted and actual values:

(1) $$L_{BCE}=-\frac{1}{N}\sum_{i=1}^{N}\left[y_{i}\log(p_{i})+(1-y_{i})\log(1-p_{i})\right],$$

where *N* represents the total number of pixels in the input image, *y_i_* represents the actual value of the *i*-th pixel, and *p*_i_ represents the predicted probability that the *i*-th pixel belongs to the left ventricular region.

### 2.4. Evaluation Metrics

The model’s performance in predicting the left ventricular region was evaluated using the DSC, which measures the overlap between the model-predicted region and the region manually annotated by human experts. DSC is a similarity metric commonly used to compute the similarity between two regions, with values ranging from 0 to 1. The higher the DSC is, the better the segmentation performance. The DSC was calculated by

(2) $$\mathrm{DSC}(A,B)=\frac{2\left|A\cap B\right|}{\left|A\right|+\left|B\right|},$$

where *A* represents the left ventricular region in a TEE A4CV image predicted by the model, and *B* represents the left ventricular region in the same TEE A4CV image manually annotated by human experts.

The Intersection over Union (IoU) was also employed to evaluate the similarity between the left ventricular region predicted by the model and the manually annotated region provided by human experts. IoU values range from 0 to 1, with higher values indicating a closer match between the predicted and annotated regions. The IoU was calculated by

(3) $$\mathrm{IoU}(A,B)=\frac{\left|A\cap B\right|}{\left|A\cup B\right|}$$

### 2.5. Statistical Analysis

The Kruskal–Wallis test [27] was used to determine whether the DSC values of UNeXt, U-Net, TransUNet, and Attention U-Net on the test set exhibited statistically significant differences. A significance level of *p* < 0.05 was considered a significant difference in DSC. Statistical analyses were conducted using IBM SPSS Statistics 27 (IBM Corp., Armonk, NY, USA).

## 3. Results

**Figure 9:**
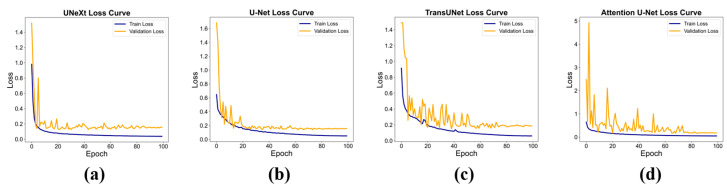
The loss on the training set (Train Loss) and validation set (validation loss) as a function of the training epochs for various deep learning models: UNeXt (**a**), U-Net (**b**), TransUNet (**c**), Attention U-Net (**d**).

Figure 9 shows the loss curves for UNeXt, U-Net, TransUNet, and Attention U-Net on both the training and validation sets. The loss values of all four deep neural networks steadily decreased on the training set and eventually stabilized. Similarly, the loss curves on the validation set closely followed those of the training set, gradually decreasing and ultimately converging. Before the 60th epoch, the validation loss curves exhibited varying degrees of fluctuation across the four networks. UNeXt showed the smallest fluctuation, while Attention U-Net experienced the largest, likely due to the limited dataset size. Although the dataset contained 3000 TEE images, the number of patients included was relatively small. After the 80th epoch and through the end of training, the loss values for all three models remained stable on both the training and validation sets, indicating convergence without signs of overfitting.

**Figure 10:**
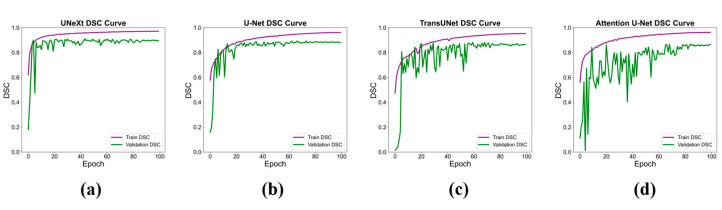
The DSC on the training set (Train DSC) and validation set (validation DSC) as a function of the training epochs for various deep learning models: UNeXt (**a**), U-Net (**b**), TransUNet (**c**), Attention U-Net (**d**). DSC: dice similarity coefficient.

Figure 10 illustrates the DSC curves for UNeXt, U-Net, TransUNet, and Attention U-Net on the training and validation sets. The DSC values of all four deep neural networks gradually increased on the training set and stabilized. The DSC curves on the validation set closely followed the training set, gradually increasing and showing no significant changes after reaching their peak. Before the 60th epoch, the validation DSC curves of the four networks exhibited varying degrees of fluctuation. UNeXt had the smallest fluctuation, while Attention U-Net had the largest, likely due to the limited dataset size.

Table 1 presents the left ventricular segmentation performance of UNeXt on the test data, compared to U-Net [5], TransUNet [12], and Attention U-Net [11]. The results showed that UNeXt achieved the highest segmentation accuracy among the four models. The Kruskal–Wallis test indicated significant differences in DSC performance on the test set among UNeXt, U-Net, TransUNet, and Attention U-Net. Pairwise comparisons indicated significant differences in DSC between Attention U-Net and UNeXt, between Attention U-Net and U-Net, between Attention U-Net and TransUNet, and between UNeXt and TransUNet. In contrast, no significant differences were identified between UNeXt and U-Net or between U-Net and TransUNet on the test set.

**Figure 11:**
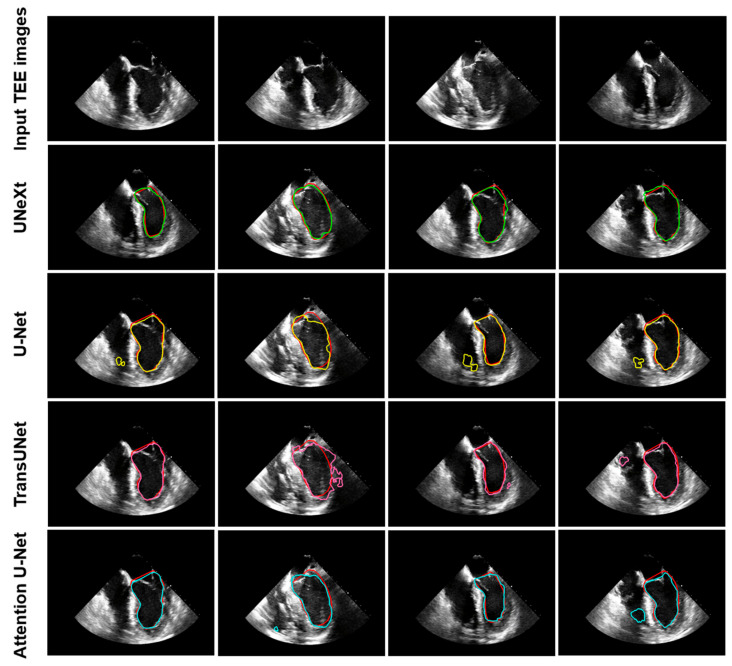
Results of TEE A4CV LV prediction by various deep learning models. Red contours represent manual LV segmentation as the reference standard. Green boundaries represent LV segmentation by UNeXt. Yellow contours represent LV segmentation by U-Net. Pink contours represent LV segmentation by TransUNet. Cyan contours represent LV segmentation by Attention U-Net. TEE: transesophageal echocardiography; LV: left ventricle.

Figure 11 shows the left ventricular segmentation results of UNeXt, U-Net, TransUNet, and Attention U-Net on the test data. Each column represents representative TEE images from different patients. The first row shows the input TEE A4CV images, and rows two to five show the segmentation results of UNeXt, U-Net, TransUNet, and Attention U-Net, respectively. It can be observed that UNeXt’s segmentation results closely match the manual annotations by human experts. All three models, U-Net, TransUNet, and Attention U-Net, tended to misidentify regions outside the left ventricle. For example, U-Net and Attention U-Net mistakenly identified areas of the right ventricle (Figure 11, third row, first, third, and fourth columns; fifth row, second and fourth columns). TransUNet showed less sensitivity in detecting the boundaries of the left ventricle (Figure 11, fourth row, third column). In contrast, UNeXt provided more accurate left ventricular identification, with better boundary delineation that more closely matched the anatomical structure of the left ventricle.

Table 2 presents the parameter counts of the different models for left ventricular segmentation. The comparison shows that the UNeXt model used in this study had significantly fewer parameters and floating point operations (FLOPs) than U-Net, TransUNet, and Attention U-Net, making it a more lightweight model.

## 4. Discussion

In this paper, a large-scale TEE dataset was constructed, and an automatic left ventricle segmentation method for the TEE A4CV based on the UNeXt deep neural network was proposed. Its performance was compared with U-Net, TransUNet, and Attention U-Net. The UNeXt deep neural network, introduced by Valanarasu et al. [26] in 2022, has the advantage of reducing network parameters while maintaining segmentation accuracy, but it had not yet been applied to TEE A4CV left ventricle segmentation. The results demonstrated that UNeXt achieved the best overall performance, with an average DSC for left ventricle segmentation of 88.60%, surpassing U-Net (87.76%), TransUNet (85.75%; *p* < 0.05), and Attention U-Net (79.98%; *p* < 0.05). Additionally, UNeXt had the smallest number of network parameters (1.47 million) and FLOPs (2.28 giga), as well as the shortest average inference time per image (141.73 ms).

TTE is the preferred cardiac ultrasound method in emergency rooms [28]. Despite its widespread use, TTE has certain limitations. Since data are collected externally on the body, it is highly dependent on the operator’s skill, leading to inconsistent image quality. In contrast, TEE, where the probe is positioned in the patient’s esophagus, allows for better guidance to the target area and more consistently produces high-quality images. TTE image acquisition is also affected by patient-related factors [29], such as obesity, mechanical ventilation, subcutaneous emphysema, and ongoing cardiopulmonary resuscitation (CPR), which may result in poor or unobtainable images. TEE, however, is not affected by factors like subcutaneous emphysema, fat tissue thickness, or surgical dressings, and can still capture high-quality images without interrupting CPR during cardiac arrest. Consequently, TEE is widely used in settings like cardiac surgery rooms and intensive care units, where it continuously monitors cardiac output and other heart function indicators in real time, assisting clinicians in adjusting surgical plans and making decisions during operations. Although deep learning techniques have been extensively studied and have demonstrated strong performance in automatic left ventricle segmentation on the TTE A4CV [15,17,18,20,21,22], there are limited studies on TEE A4CV left ventricle segmentation using deep learning [23,24]. The reason may be that constructing a large-scale TEE A4CV dataset is somewhat difficult.

As shown in Table 3, the proposed method for automatic left ventricle segmentation in the TEE A4CV based on the UNeXt deep neural network achieved an average DSC of 88.60%, which represents a 6.52% improvement compared to the Res-U-Net++ [25] used by Ahn et al. [24]. The dataset used in this study was collected from 60 patients undergoing cardiac surgery, with standard TEE A4CV videos captured from the induction of anesthesia until the conclusion of the surgery. After data preprocessing, a dataset of 3000 TEE images and corresponding labels was generated. Compared to Kang et al. [23] (120 images from nine patients) and Ahn et al. [24] (158 images), this study significantly increased both the number of patients and the volume of TEE images. However, it should be noted that the image datasets used in the studies shown in Table 3 were different, so the DSC values of different models may not be directly compared. The size and diversity of the dataset are crucial factors that influence the generalizability of the trained models. The small size of a dataset may lead to overfitting, whereas a larger and more varied dataset provides a wider range of training cases, which helps the model learn more robust features that are applicable to different situations. In this sense, the small dataset used in Kang et al. [23] and Ahn et al. [24] may limit the reliability of their models.

Although the image quality of TEE is generally clearer than that of TTE, various factors, such as patient characteristics, equipment, technical skill, and environmental conditions, can still lead to challenges. These include strong echo interference from muscle tissue, unclear TEE images, and the presence of artifacts, which posed challenges in this study.

**Figure 12:**
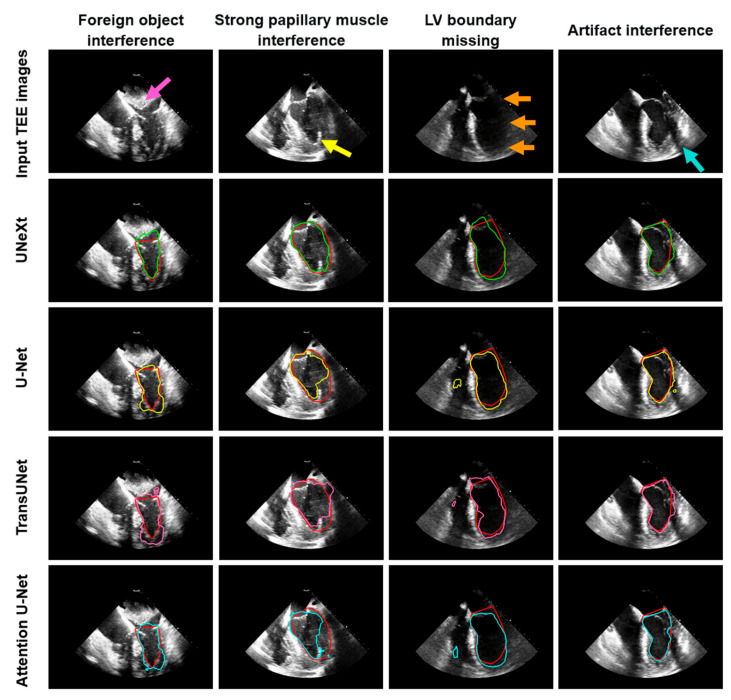
Representative LV segmentation using different deep learning models in TEE images with challenging cases: foreign object interference (column 1), strong papillary muscle interference (column 2), LV boundary missing (column 3), artifact interference (column 4). Red contours represent the manually annotated LV, serving as the ground truth. Green boundaries stand for LV segmentation by UNeXt. Yellow contours represent LV segmentation by U-Net. Pink boundaries indicate LV segmentation by TransUNet. Cyan contours indicate LV segmentation using Attention U-Net. Pink arrows indicate the foreign object. Yellow arrows indicate the papillary muscle. Orange arrows correspond to a missing LV boundary. Cyan arrows correspond to the artifact. LV: left ventricle; TEE: transesophageal echocardiography.

Figure 12 presents several challenging examples of left ventricle segmentation in TEE images. Each column represents TEE images containing different segmentation challenges. The first row shows the original TEE images input into the network models, while rows two to five display the left ventricle segmentation results from the trained UNeXt, U-Net, TransUNet, and Attention U-Net models, respectively. The boundary of the left ventricle is composed of muscular tissue, which appears as a strong echo in the ultrasound image. However, when foreign objects (lesions) are present above the left ventricle, such as in the left atrium, these also produce strong echoes in the ultrasound image. In this scenario, all four models incorrectly identified the boundary of the foreign object as the left ventricle boundary, resulting in inaccurate segmentation (Figure 12, first column). The papillary muscles within the left ventricle also exhibit strong echoes in the ultrasound image, which similarly interferes with the segmentation of the left ventricle. As shown in Figure 12 (second column), UNeXt was least affected by this interference, whereas U-Net, TransUNet, and Attention U-Net mistakenly segmented the papillary muscle as part of the left ventricle boundary, resulting in a significant deviation from the manually annotated contour. When facing incomplete left ventricle edges, U-Net and TransUNet produced segmentation results that, while not entirely accurate, closely matched the manually annotated boundaries, whereas the segmentation results of UNeXt and Attention U-Net differed more significantly (Figure 12, third column). Lastly, during ultrasound scanning, technical factors such as poor contact between the probe and the patient or insufficient coupling gel can result in artifacts, seen as low-echo bands extending from the near field to the far field in the ultrasound image. In the presence of artifacts, U-Net, UNeXt, and Attention U-Net were only slightly affected, whereas TransUNet was more sensitive to their presence (Figure 12, fourth column).

It should be noted that the left ventricle segmentation in TEE images is not the final goal in clinical settings. However, analyzing the cardiac function indices, such as the ejection fraction and cardiac output, relies on accurate segmentation of the left ventricle. Although end-to-end deep neural networks may be used to directly predict these indices, the results may lack clinical interpretability. Segmenting the left ventricle and analyzing the cardiac function indices based on the segmentation may be more in line with the clinical workflow. When each TEE A4CV image in a cardiac cycle was segmented using the proposed method, the dynamic variation in the area of the segmented left ventricle images could be used to localize the end-diastolic and end-systolic frames, which could be used to calculate the end-diastolic volume (EDV) and end-systolic volume (ESV) using methods such as the Simpson bi-plane method. The calculated EDV and ESV could be used to compute the left ventricular ejection fraction or cardiac output (together with heart rate). In sum, the proposed method may be used for fast, automatic left ventricular segmentation in TEE A4CV images, facilitating dynamic assessment of left ventricle function.

This study has limitations. First, although the constructed dataset contains 3000 TEE images, the number of patients remains relatively small. Second, while the UNeXt network was applied to the task of left ventricle segmentation on the TEE A4CV, the DSC may be increased by improving the network structure. In future work, more patients can be included and improvement on the UNeXt network (e.g., EnsUNet [30]) can be considered to further improve the model performance.

## 5. Conclusions

In this study, we constructed a large-scale TEE A4CV dataset and proposed an automatic segmentation method for the left ventricle on the TEE A4CV based on the UNeXt deep neural network, which was compared with U-Net, TransUNet, and Attention U-Net networks. A dataset consisting of 3000 TEE images from 60 patients and their corresponding labels was constructed, and the three deep neural networks were trained and validated. The results indicated that the UNeXt network yielded an average DSC of 88.60% on the test set, outperforming U-Net (87.76%), TransUNet (85.75%; *p* < 0.05), and Attention U-Net (79.98%; *p* < 0.05). Additionally, the UNeXt model had a smaller number of parameters and FLOPs (1.47 million, 2.28 giga) and shorter average inference times per image (141.73 ms) than U-Net (185.12 ms), TransUNet (209.08 ms), and Attention U-Net (201.13 ms). The average IoU of UNeXt (77.60%) was also higher than that of U-Net (76.61%), TransUNet (77.35%), and Attention U-Net (68.86%). The proposed method may serve as a new deep learning approach for automatic segmentation of the left ventricle in TEE A4CV images, with promising segmentation accuracy and reduced inference time.

## Figures and Tables

**Table 1:** Left ventricular segmentation performance by different deep learning models. DSC: dice similarity coefficient; IoU: Intersection over Union.

Deep Learning Models	DSC (%)	IoU (%)
U-Net [5] TransUNet [12]	87.76 ± 3.61 85.75 ± 5.93	76.61 ± 7.45 77.35 ± 8.50
Attention U-Net [11]	79.98 ± 9.26	68.86 ± 11.95
UNeXt [26]	88.60 ± 2.92	77.60 ± 6.33

**Table 2:** Parameters of different deep learning models. FLOPs: floating point operations.

Deep Learning Models	# of Model Parameters (million)	Inference Time for a Single Image (ms)	FLOPs (giga)
U-Net [5]	7.85	185.12	218.57
TransUNet [12]	79.71	209.08	272.24
Attention U-Net [11]	34.88	201.13	265.92
UNeXt [26]	1.47	141.73	2.28

**Table 3:** The comparison of LV segmentation performance among different methods using TEE data. LV: left ventricle; TEE: transesophageal echocardiography; DSC: dice similarity coefficient.

Authors	Year	Deep Learning Models	Data	DSC (%)
Kang, et al. [23]	2023	Variation of U-Net [5]	128 TEE images of 9 patients	89.90
Ahn, et al. [24]	2023	Res-U-Net++ [25]	158 TEE images (number of patients not described)	82.08
Ours	2024	UNeXt [26]	3000 TEE images of 60 patients	88.60

## Data Availability

Data are unavailable due to ethical restrictions.
